# Isoliquiritigenin prevents hyperglycemia-induced renal injuries by inhibiting inflammation and oxidative stress via SIRT1-dependent mechanism

**DOI:** 10.1038/s41419-020-03260-9

**Published:** 2020-12-07

**Authors:** Xiaozhong Huang, Yujuan Shi, Hongjin Chen, Rongrong Le, Xiaohua Gong, Ke Xu, Qihan Zhu, Feixia Shen, Zimiao Chen, Xuemei Gu, Xiaojun Chen, Xiong Chen

**Affiliations:** 1 Department of Pediatric Surgery, The Second Affiliated Hospital and Yuying Children’s Hospital, Wenzhou Medical University, Wenzhou, China; 2 Department of Endocrinology, Jiangshan People’s Hospital, Jiangshan, Zhejiang China; 3 Department of Pathology, School of Basic Medical Sciences, Nanjing Medical University, Nanjing, China; 4 Eye Hospital and School of Ophthalmology and Optometry, Wenzhou Medical University, Wenzhou, Zhejiang China; 5 Department of Endocrinology, the First Affiliated Hospital, Wenzhou Medical University, Wenzhou, Zhejiang China

**Keywords:** Pharmacology, Molecular biology

## Abstract

Diabetic nephropathy (DN) as a global health concern is closely related to inflammation and oxidation. Isoliquiritigenin (ISL), a natural flavonoid compound, has been demonstrated to inhibit inflammation in macrophages. Herein, we investigated the effect of ISL in protecting against the injury in STZ-induced type 1 DN and in high glucose-induced NRK-52E cells. In this study, it was revealed that the administration of ISL not only ameliorated renal fibrosis and apoptosis, but also induced the deterioration of renal function in diabetic mice. Mediated by MAPKs and Nrf-2 signaling pathways, respectively, upstream inflammatory response and oxidative stress were neutralized by ISL in vitro and in vivo. Moreover, as further revealed by the results of molecular docking, sirtuin 1 (SIRT1) binds to ISL directly, and the involvement of SIRT1 in ISL-mediated renoprotective effects was confirmed by studies using in vitro models of SIRT1 overexpression and knockdown. In summary, by reducing inflammation and oxidative stress, ISL has a significant pharmacological effect on the deterioration of DN. The benefits of ISL are associated with the direct binding to SIRT1, the inhibition of MAPK activation, and the induction of Nrf-2 signaling, suggesting the potential of ISL for DN treatment.

## Introduction

In recent years, diabetes mellitus has become a global health concern. As a major characteristic of diabetic patients, the abnormally prolonged hyperglycemia can lead to a series of complications^1^. Among the most common and severe diabetic complications is diabetic nephropathy (DN), which has been identified as the primary reason for persistent kidney disease and terminal renal failure^2^. The pathological symptoms of DN include the damage of the renal tubular epithelial cells, glomerular sclerosis and apoptosis, inflammatory infiltration of nephrocytes, and renal tubular interstitial fibrosis^3^. Emerging evidence has suggested that inflammation^4^ and oxidative stress^5^ are two significant contributory factors in the progression of DN. As confirmed by Chen et al., antiinflammatory compounds are effective in attenuating hyperglycemia-induced IL-6 and TNF-α increase in DN^6^. As the significant transcription factors in inflammation, nuclear factor-κB and activator protein-1 (AP-1), considered as two poor indicators of DN individuals, are also involved in the development of DN^7,8^. In addition, the increased expression of antioxidant genes (e.g., Nrf-2, HO-1, and NQO-1) has been demonstrated in many studies as effective in preventing the oxidative damage caused by hyperglycemia^9,10^. Therefore, the agents capable of reducing inflammation and oxidation might shed light on the molecular mechanism underlying the progression of the disease, thus indicating the future direction of developing the drugs used to treat DN.

Isoliquiritigenin (ISL), a natural flavonoid compound isolated from *Glycyrrhiza uralensis* (licorice), *Sinofranchetia chinensis*, and *Dalbergia odorifera*, is capable to perform a variety of different pharmacological and biological activities, for example, antiinflammatory^11^, antioxidant^12^, and antitumor activities^13^. As indicated by the lipopolysaccharide (LPS)-induced models of inflammation, ISL can produce a significant antiinflammatory effect^14^. Recently, studies have been conducted to demonstrate the effectiveness of ISL in protecting against diabetes-related diseases, such as, insulin sensitivity^15^, diabetic neuropathy^16^, and adipose tissue inflammation^17^. However, the underlying mechanism behind the effect of ISL on DN and the targets of ISL remains unclear. Flavonoid compounds are effective in mitigating hyperglycemia-induced diabetic complications by enhancing the expression of the sirtuin family, with the silent information regulator 2 homolog 1 (SIRT1) in particular, as shown in more and more studies^18–20^. SIRT1 is a NAD+‐dependent histone deacetylase that is related to various biological activities, for example, DNA repair, oxidation, and inflammation. In diabetic neuropathy, ISL produces an alleviative effect on mitochondrial impairment by activating SIRT1^16^.

Thus, we hypothesized that ISL could inhibit the hyperglycemia-induced inflammatory and oxidative damages caused by the development and progression of DN by restoring SIRT1. In order to test this hypothesis, we investigated the protective effects of ISL by streptozotocin (STZ) injection in vivo and high glucose (HG)-stimulated renal epithelial cell line in vitro. According to our findings, ISL mitigated the functional and pathologic damages by significantly suppressing inflammation and oxidative stress both in vivo and in vitro. Besides, we testified the role of SIRT1 in mediating the mechanisms for the renal protective effects of ISL in DN. These results shed light on ISL as a potential solution to DN treatment.

## Results

### ISL could reduce hyperglycemia-induced renal histological abnormalities and biochemical indicators increase

**Fig. 1:**
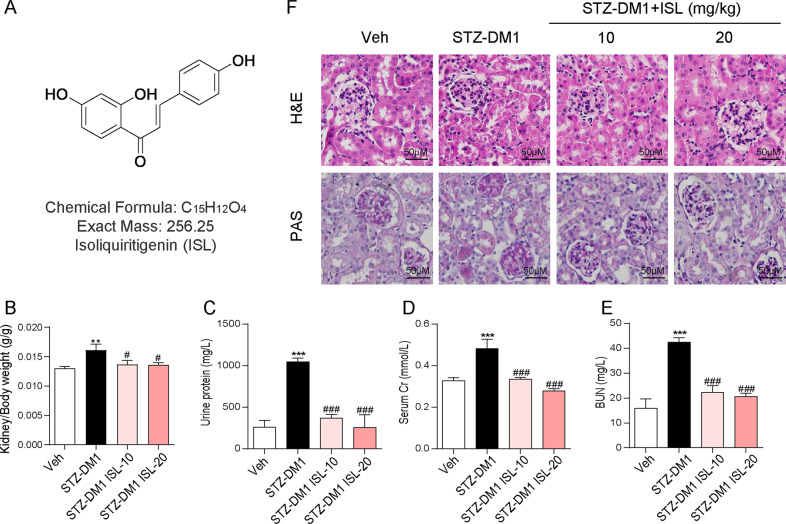
ISL attenuated hyperglycemia-induced renal histological abnormalities and biochemical indicators increase. Diabetes mellitus was induced by streptozotocin (STZ), which dissolved in 0.1 mol/L sodium citrate buffer (pH 4.5) for intraperitoneal injection at 50 mg/kg/day that lasted 5 days. The mice with fasting-blood glucose levels exceeding 12 mM were considered diabetic. Diabetic mice were then subject to oral treatment using ISL (10 or 20 mg/kg) or vehicle (0.5% CMC-Na) by gavage every other day for 12 weeks (*n* = 7 in each group). 24 h before the conclusion of experiment, mice urine was collected. After killing, kidney tissues of the mice were collected and blood samples centrifuged to collect serum for further analysis. **a** The structure of ISL. **b** The kidney/body weight ratio. **c** Total protein concentration in urine. **d** The level of serum creatinine. **e** The level of serum BUN. **f** Representative images for H&E staining and PAS staining using the formalin-fixed renal tissues as described in Methods and materials (×400 magnification) (A minimum of five mice in each group were used for analysis. **P < 0.01, ***P < 0.001 vs control (Veh); ^#^*P* < 0.05, ^###^*P* < 0.001 vs STZ1-DM).

In order to determine whether ISL could affect hyperglycemia-induced renal injury in vivo, STZ-induced type 1 mice model (T1DM) were established for ISL (10 or 20 mg/kg) or vehicle treatment for a 8-week period. First, we investigated the ratio of kidney weight to body weight, which is a marker of renal swelling and injury. According to Fig. 1b, the ratio was higher in the group with streptozotocin injection (STZ-DM1) than in the Vehicle group, suggesting that hyperglycemia had led to renal swelling. However, ISL treatment reversed this change. Similarly, kidney function makers were significantly increased in the STZ-DM1 group, such as urine protein, serum creatinine (SCr), and blood urea nitrogen (BUN) in serum. By contrast, these biomarkers maintained stability in the group of ISL-treated diabetic mice (Fig. 1c–e). Then, through H&E staining, it was found out that ISL mitigated hyperglycemia-induced renal injury in the diabetic mice, such as glomerulus expansion, glomerulosclerosis, the narrowing of capillary lumen, the increase in size of diffused mesangial matrix and peripheral capillaries of thick stiff wall (Fig. 1f). According to Periodic acid–Schiff (PAS) staining results, PAS-positive materials (purple plaques) were significantly increased, indicating the accumulation of glycogen in glomeruli in diabetic mice. Comparatively, in the diabetic mouse group treated with ISL, as the drug concentration gradient was on the rise, the pathological changes in the glomeruli showed a significant reduction (Fig. 1f), thus confirming that ISL can contribute to mitigating STZ-induced renal injury.

### ISL suppressed hyperglycemia-induced renal fibrosis and apoptosis

**Fig. 2:**
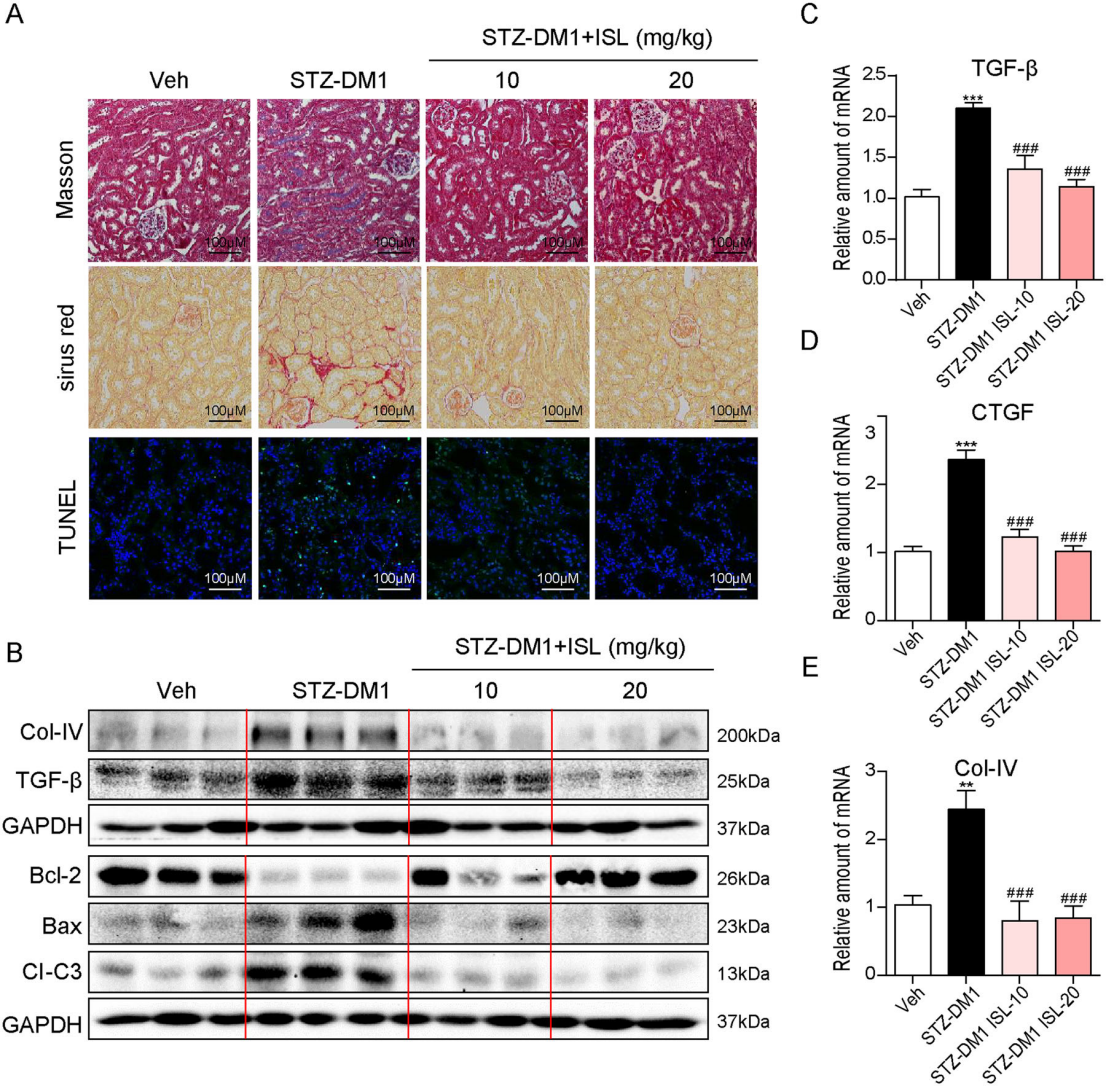
ISL suppressed hyperglycemia-induced renal fibrosis and apoptosis. **a** Representative images for Masson staining, Sirius red staining, and TUNEL staining using the formalin-fixed renal tissues as specified in Methods and materials (×200 magnification). **b** Western blot analysis for the protein expression of COL-IV, TGF-β, BCL-2, BAX, and CI-C3 in three kidney tissue extracts, each from a different mouse. **c**–**e** The mRNA expression of pro-fibrotic genes TGF-β, CTGF, and COL-IV in the kidney tissues was determined by real-time qPCR (A minimum of five mice per group were used for analysis. **P < 0.01, ***P < 0.001 vs control (Veh); ^###^*P* < 0.001 vs STZ1-DM).

So far, there have been increasing evidence, suggesting that hyperglycemia can cause renal fibrosis and apoptosis, which are significant to the pathogenesis of diabetic kidney injury. In this study, we established whether ISL could reduce pathological changes in diabetic mice. Renal fibrosis and the accumulation of collagen were documented in the STZ-DM1 group through Masson staining and Sirius Red staining. As shown in Fig. 2a, either the 10 or 20 mg/kg dose of the ISL treatment significantly inhibited hyperglycemia-induced fibrosis in diabetic mice. Meanwhile, terminal deoxynucleotidyl transferase dUTP nick end labeling (TUNEL) staining revealed that the STZ-DM1 group induced renal cell apoptosis (positive TUNEL staining area). However, similar changes were not observed in the diabetic mice pretreated with ISL (10 or 20 mg/kg) (Fig. 2a). Furthermore, our investigation was conducted into not only two representative pro-fibrotic proteins Col-IV and transforming growth factor-β (TGF-β), but also the critical pro-apoptotic proteins BAX, BCL-2, and cleave-caspase-3 (Cl-C3) in renal tissues. As shown in Fig. 2b, the STZ-DM1 group showed an increasing expression of pro-fibrotic and pro-apoptotic proteins. Consistent with the pathological findings, ISL treatment contributed to reversing the diabetes-induced overexpression of pro-fibrotic and pro-apoptotic proteins. Notably, a similar protective effect to the treatment of ISL at 10 mg/kg was manifested in the diabetic mice treated with 20 mg/kg of ISL. Moreover, renal tissues were also tested for the mRNA levels of TGF-β, CTGF, and Col-IV. As indicated by the results, the treatment with ISL at a 10 or 20 mg/kg dose significantly inhibited hyperglycemia-induced fibrosis gene transcription (Fig. 2c–e).

### ISL improved hyperglycemia-induced renal inflammation and oxidation

**Fig. 3:**
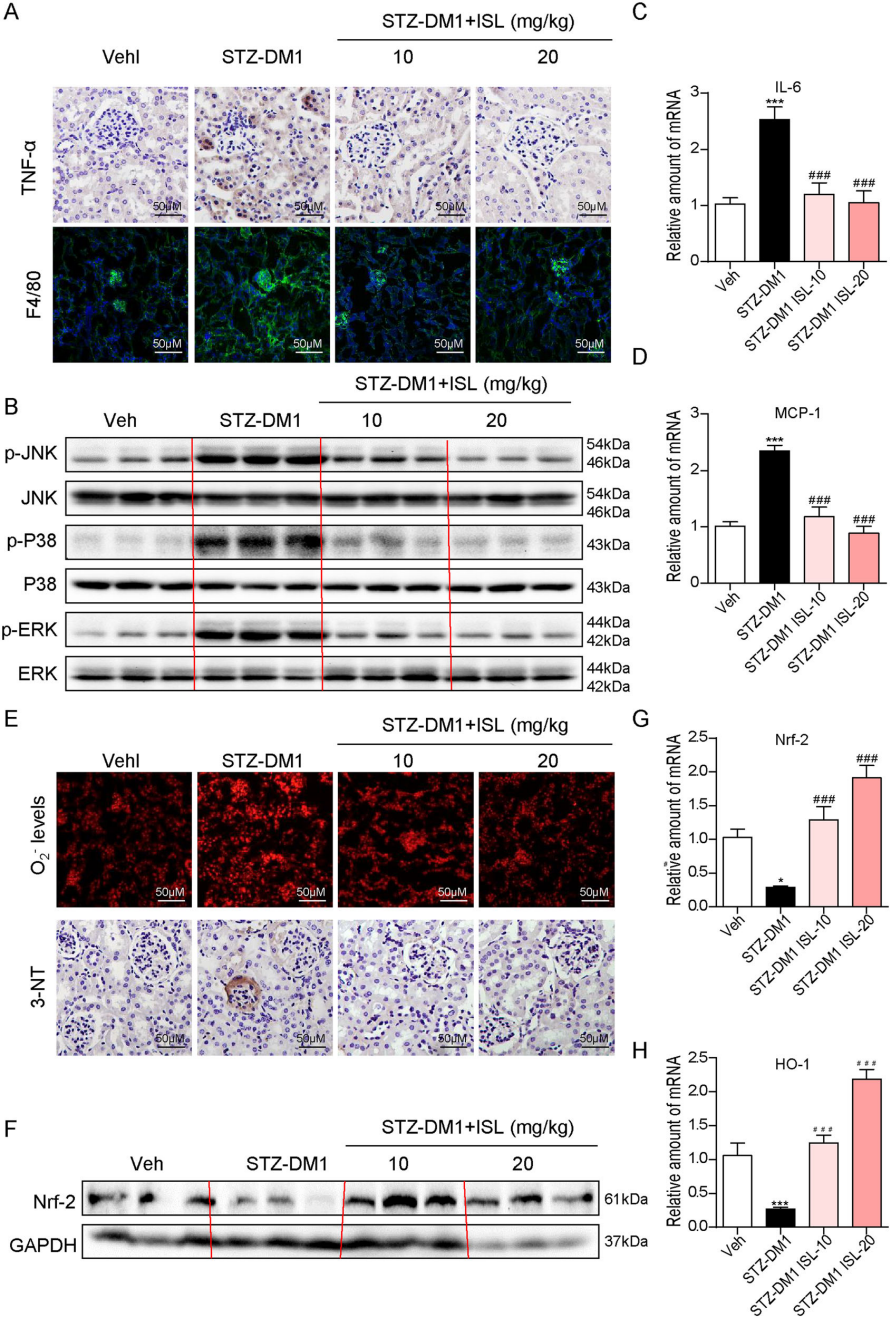
ISL improved hyperglycemia-induced renal inflammation and oxidative stress. **a** Representative images for TNF-α and F4/80 immunohistochemistry staining using the formalin-fixed renal tissues as specified in Methods and materials (×400 magnification). **b** Western blot analysis for the protein expression of p-JNK, JNK, p-p38, p38, p-ERK, and ERK in three kidney tissue extracts, each from a different mouse. **c**, **d** The mRNA expression of pro-inflammatory genes IL-6 and MCP-1 in the kidney tissues was determined by real-time qPCR. **e** Representative images for DHE and 3-NT immunohistochemistry staining using the formalin-fixed renal tissues as specified in Methods and materials (×400 magnification). **f** Western blot analysis for the protein expression of Nrf-2 and GAPDH in three kidney tissue extracts, each from a different mouse. **g**, **h** The mRNA expression of anti-oxidation genes Nrf-2 and HO-1 in the kidney tissues was determined by real-time qPCR (A minimum of five mice per group were used for analysis. *P < 0.05, ***P < 0.001 vs control (Veh); ^###^*P* < 0.001 vs STZ1-DM).

Up to now, there have been cumulative evidence, suggesting that inflammatory response and oxidative stress are two significant factors in diabetic complications, especially in DN. In addition, it has been reported that ISL is an excellent antiinflammatory and antioxidant compound. Based on these findings, the antiinflammatory and antioxidative activities of ISL in diabetic kidneys were investigated in this study. First, cytokine release and macrophage infiltration were observed using immunohistochemistry method. According to Fig. 3a, the hyperglycemia-induced expression of TNF-α and F4/80 in the renal tissues of diabetic mice was suppressed by ISL treatment. As indicated by the further test of pro-inflammatory genes, the hyperglycemia-induced overexpression of IL-6 and MCP-1 in renal tissues of diabetic mice could be effectively downregulated by ISL treatment (10 or 20 mg/kg) (Fig. 3c, d). Macrophage is critical to the process of inflammatory response. It was examined whether ISL could eliminate the inflammation in HG-stimulated macrophages. ISL could significantly inhibit the HG-induced increases of mRNA levels of TNF-α (supplementary Fig. 1A) and IL-6 (supplementary Fig. 1B). In addition, ISL suppressed TNF-α and IL-6 increasing, which induced by HG (supplementary Fig. 1C, D). The activation of components of MAPKs family has been widely recognized as an essential pathway during hyperglycemia-induced inflammatory cascade. As shown in Fig. 3b, ISL significantly inhibited the increased phosphorylation of p38, ERK, and JNK caused by hyperglycemia. However, there was no significant reduction to ERK phosphorylation in the ISL-10 mg/kg group. Thus, it was speculated that the antiinflammatory effect of ISL could be achieved by inhibiting MAPKs phosphorylation.

Then, an examination was conducted on the changes to oxidative stress in diabetic mice with or without the administration of ISL. As shown in the O2^−^ production by dihydroethidium (DHE) staining (Fig. 3e), there were more cells with clearly up-scaled O2^−^ production found in STZ-DM1 group than in the control group and ISL-treated group. Meanwhile, the expression of 3-NT was tested by immunohistochemistry staining, which shows that the raised 3-NT levels in diabetic renal tissues were significantly inhibited by ISL regulation (Fig. 3e). Furthermore, ISL prevented HG-induced decrease of SOD activity levels in kidney tissues (supplementary Fig. 2A) and NRK-52E cells (supplementary Fig. 2B). Nrf-2 was identified as the regulator of cellular resistance to oxidants and HO-1 was the downstream molecule of Nrf-2. ISL increased the mRNA levels of Nrf-2 and HO-1, both of which were inactivated by hyperglycemia (Fig. 3g, h). Consistently, ISL significantly neutralized the protein level of Nrf-2, which was reduced in STZ-DM1 group (Fig. 3f). To sum up, our findings indicated that ISL inhibited MAPKs-mediated signaling pathway and activated Nrf-2-mediated signaling pathway, thus enhancing renal inflammatory response and the accumulation of ROS in the renal tissues of diabetic mice.

### ISL reduced HG-induced renal inflammation, oxidative stress, fibrosis, and apoptosis in NRK-52E cells

**Fig. 4:**
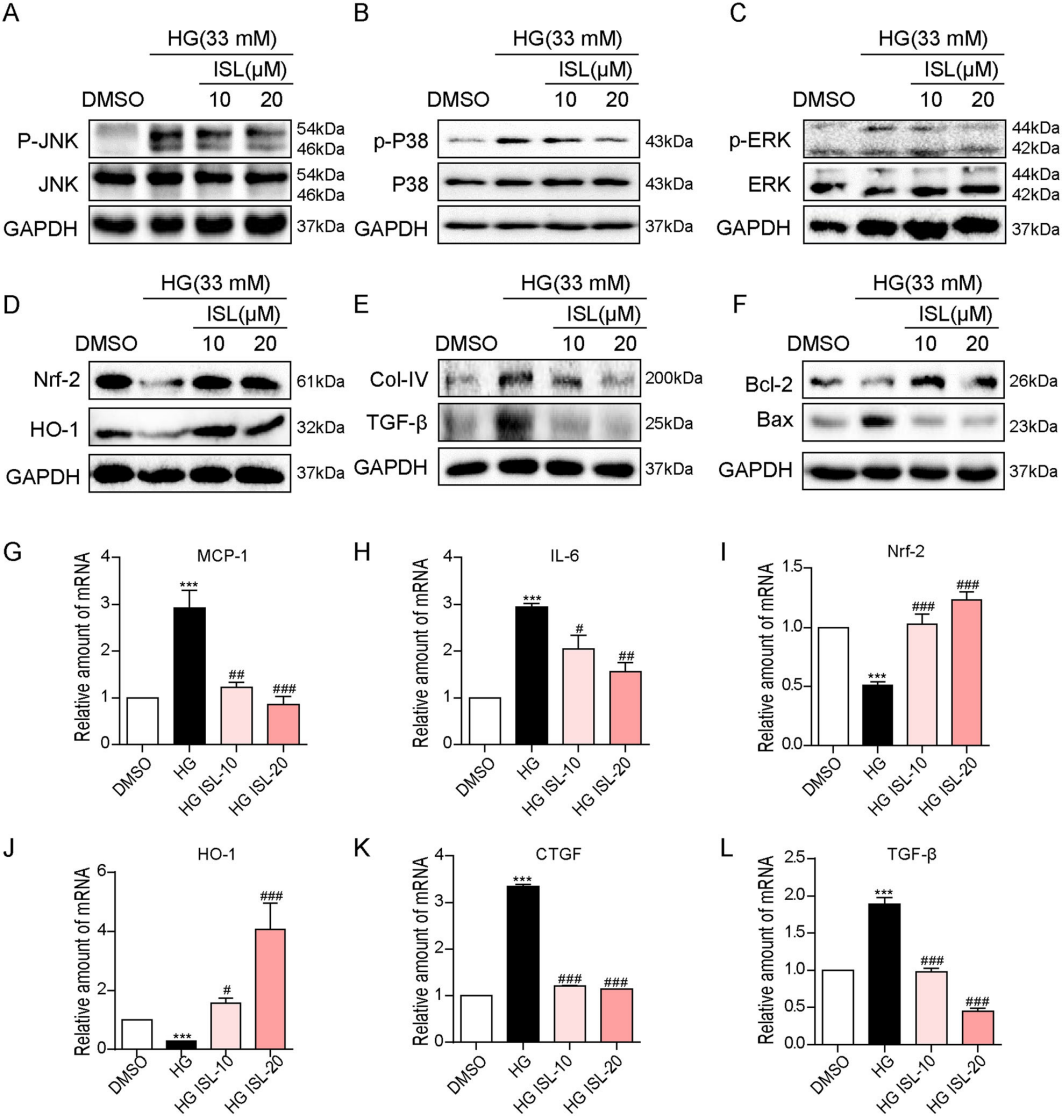
ISL reduced HG-induced inflammation, oxidative stress, fibrosis, and apoptosis in NRK-52E cells. NRK-52E cells (1 × 10^6^) were challenged with HG for the indicated time and subjected to 1 h pre-treatment with ISL (10 or 20 μM). **a**–**c** After 1 h HG-stimulated, western blot analysis was performed to investigate MAPK activation, showing the phosphorylation of p38 **a**, ERK **b**, and JNK **c** with the corresponding total protein as a loading control. **d** 12 h after HG-stimulated, western blot analysis was performed to study the expression of Nrf-2 and HO-1 with GAPDH as a loading control. **e**, **f** 36 h after HG-stimulated, western blot analysis was carried out to study the expression of pro-fibrosis genes, COL-IV and TGF-β, or apoptosis-related genes, BAX and BCL-2, with GAPDH as a loading control; **g**–**l** The mRNA levels of MCP-1 and IL-6 in 6 h after HG-stimulated **g**, **h**; or Nrf-2 and HO-1 in 4 h after HG-stimulated **i, j**; or CTGF and TGF-β in 12 h after HG-stimulated **k**, **l** were detected by RT-qPCR. Bars represented means ± SEMs of four independent experiments (***P < 0.001 vs DMSO group; ^#^*P* < 0.05, ^##^*P* < 0.01, ^###^*P* < 0.001 vs HG group).

As revealed by the in vivo experiment, ISL could mitigate not only functional but also pathological damages associated with DN. Therefore, to confirm the protective effect of ISL and to explore the relevant mechanisms in vitro, *rattus norvegicus* kidney cells (NRK-52E) were used to assess the inflammatory cytokines and components of MAPKs signaling pathway. ISL treatment suppressed the HG-induced increase in the phosphorylation levels of JNK, p38 and ERK depending on dosage (Fig. 4a–c), followed by a decrease in the mRNA levels of inflammatory cytokines, IL-6 and MCP-1 (Fig. 4g, h). Afterwards, the results showed that Nrf-2 expression was suppressed by the HG stimulation in NRK-52E cells (Fig. 4d, l) in terms of mRNA and protein, along with declined mRNA expression of HO-1 (Fig. 4j). However, the inhibition of Nrf-2 and HO-1 expressions were reversed by administration of ISL treatment (Fig. 4d, i, j).

We then evaluated effects of ISL on attenuating the pro-fibrotic and pro-apoptotic responses in NRK-52E cells. observed in the diabetic mouse model. ISL significantly attenuated HG-induced increased expression of COL-IV (Fig. 4e), TGF-β and CTGF (Fig. 4k, l). In addition, ISL readily reduced the HG-induced changes in the protein levels of BCL-2 and BAX (Fig. 4F). Furthermore, a test was conducted on the protective effects of ISL on TNF-α-induced cell death and oxidative stress in NRK-52E cells. As indicated by CCK-8 assay, ISL significantly improved cells survival which was reduced by TNF-α-induced cells death in NRK-52E cells (supplementary Fig. 3A). According to western blot assay, not only did ISL reverse the TNF-α-induced inhibition of antiapoptotic Bcl-2 expression, it also reduced the enhancement of pro-apoptotic Bax expression in NRK-52E cells (supplementary Fig. 3B). Moreover, according to supplementary Fig. 3c, TNF-α-induced reduction of SOD activity was reversed by ISL.

### The binding mode between SIRT1 and ISL

**Fig. 5:**
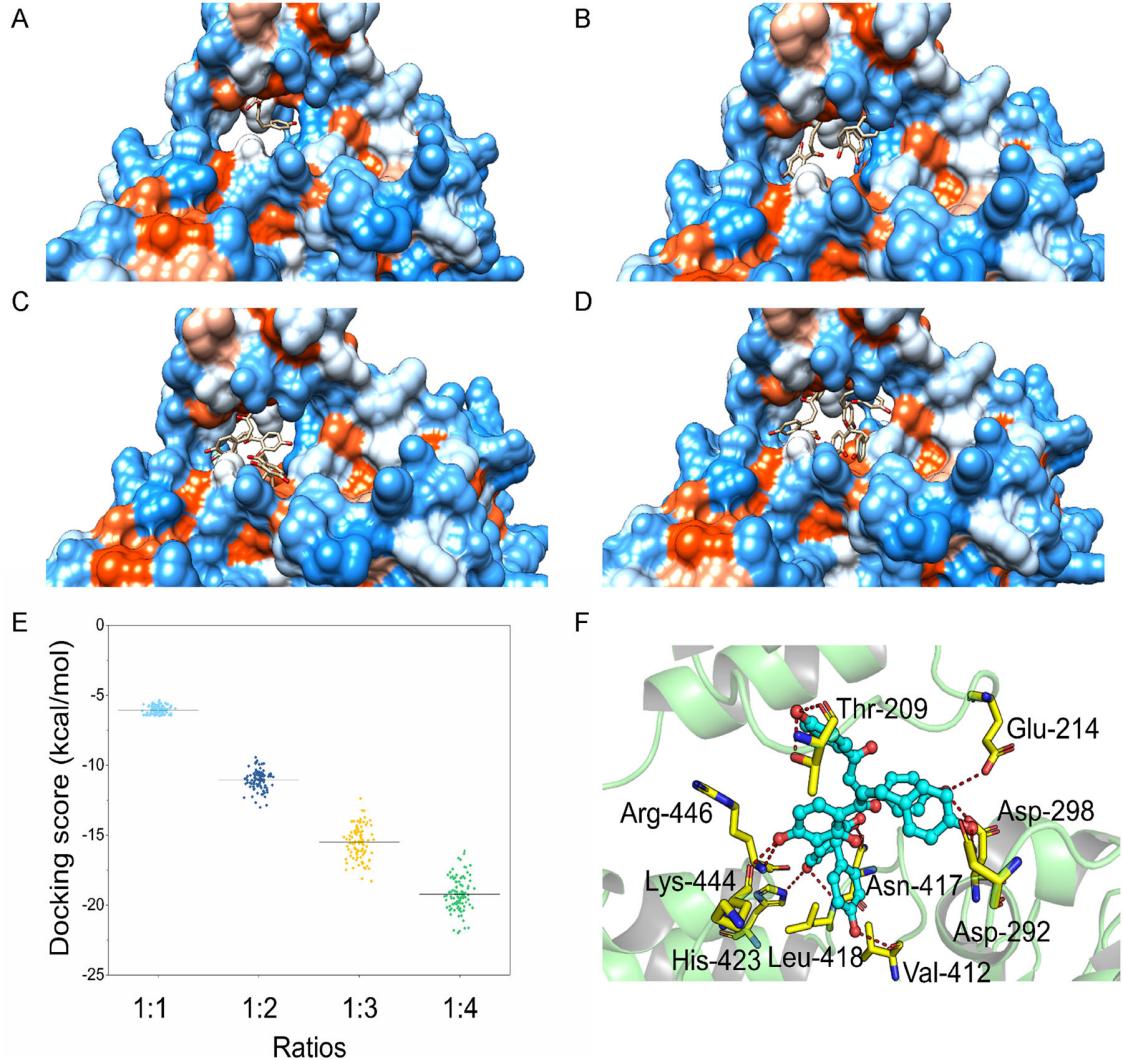
The binding mode between SIRT1 and ISL. Binding mode analysis between ISL and SIRT1 with different ratios. **a** 1:1; **b** 1:2; **c** 1:3; **d** 1:4. **e** The docking score distributions for each ratio. **f** A detailed view of SIRT1: ISL at a binding ratio of 1:3.

To predict the binding mode between ISL and SIRT1, molecular docking was performed at a binding ratio of 1:1, 1:2, 1:3, and 1:4, respectively. As shown in Fig. 5a–d, a maximum of three molecules of ISL can be appropriately bound in the binding site of SIRT1. With four molecules of ISL, one of the ISL exhibited an abnormal binding mode that appeared to have escaped the SIRT1-binding site. Then, an analysis was conducted of the docking score distributions at each ratio. As shown in Fig. 5e, an increasing binding ratio is matched with a higher amount of binding free energy, with the optimal binding free energy at a 1:4 binding ratio. However, as there is an unnatural binding mode for the 1:4 ratio in the binding site of SIRT1, the 1:3 ratio was taken as an alternative to structural analysis. According to Fig. 5f, the residues of Thr-209, Glu-214, Asp-292, Asp-298, Val-412, Asn-417, Leu-418, His-423, Lys-444, and Arg-446 formed a hydrogen bond with the three molecules of ISL. Among these important residues, Asp-292 and Asp-298 were found as well in the crystal structure of SIRT1/resveratrol complex at a ratio of 1:3, thus enabling a resveratrol hydrogen bond with SIRT1^21^. Thus, the molecular docking results indicate a possibility that the three molecules of ISL might bind to the binding site of SIRT1, thus forming a stable complex.

### ISL abrogated HG-induced inflammatory and oxidative injuries in NRK-52E cells via SIRT1

**Fig. 6:**
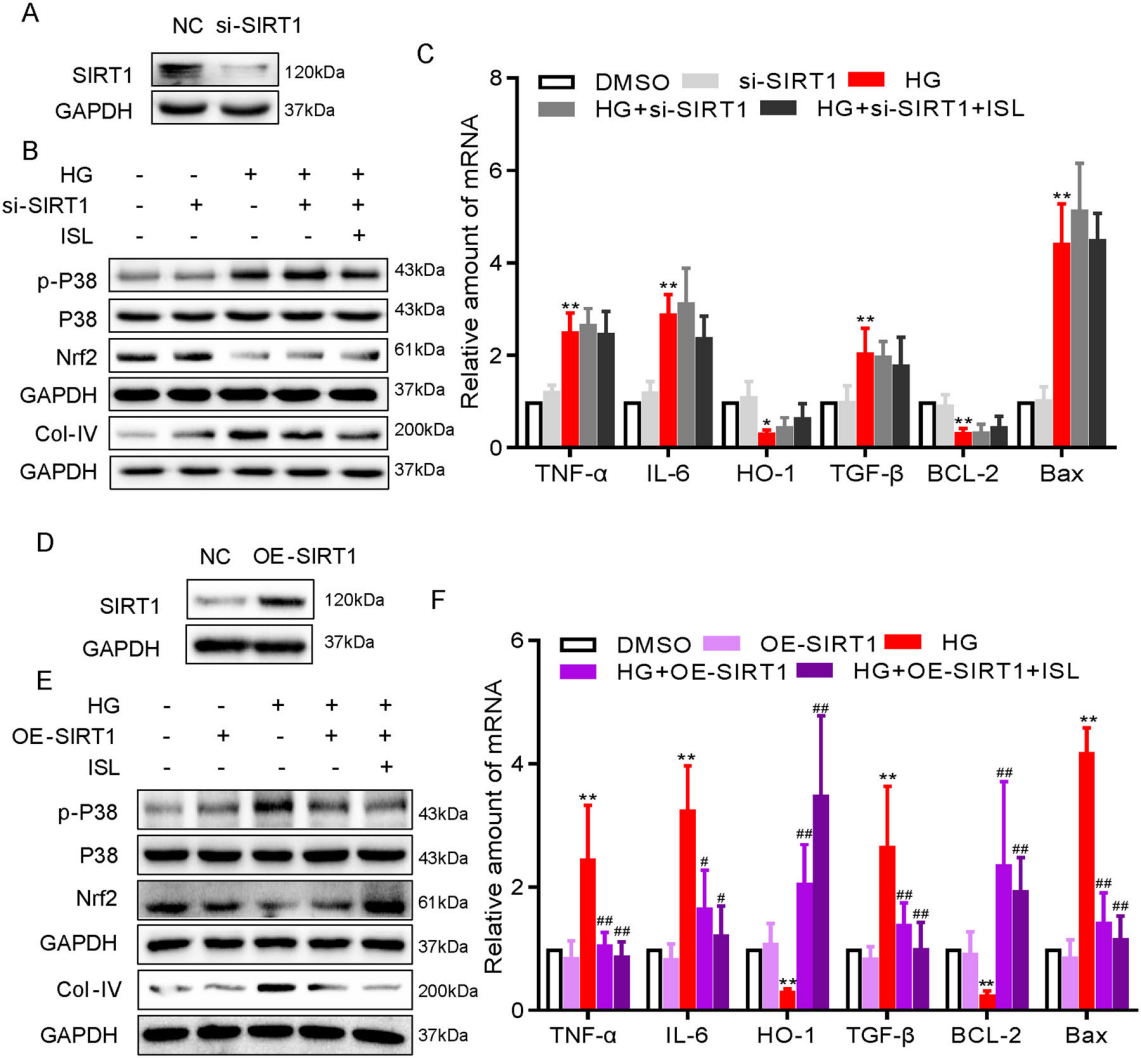
ISL inhibited HG-induced inflammatory and oxidative injuries in NRK-52E cells via SIRT1. NRK-52E cells (1 × 10^6^) were challenged with HG for the indicated time and subjected to 1 h pre-treatment with ISL (10 μM). **a** NRK-52E cells were transfected with siRNA against SIRT1. Control cells were transfected with negative control siRNA. Western blot was used to determine knockdown efficiency. **b** Immunoblot analysis of p-p38, p38, Nrf-2, and Col-IV following SIRT1 knockdown. **c** The mRNA expressions of TNF-α, IL-6, HO-1, TGF-β, Bcl-2, and Bax was determined by RT-qPCR at the same time as in Fig. 4. **d** NRK-52E cells were transfected with cDNA plasmids encoding SIRT1. Control cells were transfected with empty vector. Western blot was used to determine SIRT1 expression. **e** Immunoblot analysis of p-p38, p38, Nrf-2, and collagen IV following SIRT1 overexpression. **f** The mRNA expressions of TNF-α, IL-6, HO-1, TGF-β, Bcl-2, and Bax was determined by real-time qPCR at the same time as in **c** (*P < 0.05, **P < 0.01 vs DMSO group; ^#^*P* < 0.05, ^##^*P* < 0.01 vs HG group).

NRK-52E cells were transfected with SIRT1-targeting siRNA and SIRT1-expressing plasmids to investigate the protective effects of ISL as mediated by SIRT1. In SIRT1-silencing NRK-52E cells, ISL failed to reverse the HG-induced phosphorylation of p38 and inactivation of Nrf-2 (Fig. 6d, e). Then, the inhibitions of ISL on the HG-induced increase in the expression of pro-inflammatory cytokines (TNF-α and IL-6), pro-fibrotic (COL-IV and TGF-β), and pro-apoptotic (Bcl-2 and Bax) markers was reduced by SIRT1-silencing (Fig. 6e, f). Conversely, the overexpression of SIRT1 inhibited the HG-induced phosphorylation of p38 and the inactivation of Nrf-2 (Fig. 6d, e). Consequently, HG-induced TNF-α and IL-6 showed no significant increase after the overexpression of SIRT1 in NRK-52E cells (Fig. 6f). Subsequently, the overexpressed SIRT1 reduced HG-induced matrix protein expression and apoptosis significantly, as evidenced by the declining level of Col-IV at protein level, as well as TGF-β, Bcl-2 and Bax at mRNA levels (Fig. 6e, f). Collectively, these results demonstrated that ISL engages SIRT1 in inhibiting HG-induced inflammatory and oxidative injures in NRK-52E cells.

## Discussion

DN poses a severe threat to human health worldwide with its high morbidity and mortality. Increasing evidence suggests that under persistent hyperglycemia conditions, inflammatory response^22^, and oxidative stress^23^ can exceed the normal level, which have been identified as two important mechanisms behind the occurrence and progression of DN in STZ-induced T1DM. In this study, an examination was conducted on the pharmacological activity of a natural compound named ISL in the development of DN in vivo and in vitro. The renoprotective effects of ISL were documented with inhibited inflammation and oxidative stress. It was also demonstrated that the SIRT1 is a significant target mediating the renoprotective effects of ISL on STZ-induced T1DM.

As suggested by the hyperglycemia-triggered activation of inflammation signaling and macrophage infiltration, inflammatory response is also essential to how DN develops^24^. Specifically, the expression of inflammatory cytokines (IL-1β, IL-6, and TNF-α) and cell adhesion molecules (ICAM-1 and VCAM-1) in the renal tissue was significantly enhanced by chronic hyperglycemia, followed by the acceleration of structural and functional impairments^25^. Previously, the antiinflammatory role of ISL in an acute lung injury model was tested using LPS. According to the research results, compared with the LPS group, ISL treatment reduced lung inflammation, thus evidencing the effectiveness of ISL in reducing inflammation^14^. Notably, it was also revealed in this study that ISL suppressed the expression of inflammatory cytokines and cell adhesion molecules in the renal tissues of type 1 diabetic mice, as well as on macrophage infiltration (Fig. 3a, c, d). The experiment on the NRK-52E cells substantiated that ISL inhibited the expression of the inflammatory cytokines caused by HG in vitro (Fig. 4g, h). Based on these findings, it could be concluded that ISL is capable to protect against DN by blocking the inflammatory cascade.

At present, it is well known that MAP kinases family, inclusive of ERK, JNK, and p38, act as canonical intracellular signaling pathway of inflammatory and immune signaling pathway. Moreover, MAPKs was previously identified as the upstream kinases responsible for AP-1 and then confirmed as capable to mediate inflammatory cascades^26^. Also, it is widely recognized that MAPKs signaling pathway can be activated by HG or diabetes, thus contributing to DN^27,28^. It has been reported in a number of studies that a variety of natural products, including tripterygium^29^, ginsenoside^30^, and oridonin^31^, could inhibit inflammatory response in DN through the downregulation of MAPKs pathway. Herein, it was demonstrated that ISL inhibited the MAPKs pathways activated in STZ-induced DN and HG-challenged NRK-52E cells (Figs. 3b, 4a–c). Therefore, it is justified to infer that ISL reduces inflammation by mitigating diabetes-induced renal injuries through the inhibition of MAPKs signaling pathway.

In addition to inflammatory response, it has also been demonstrated in existing studies that the accumulation of ROS can cause severe damages to DN, including renal fibrosis and apoptosis^32,33^. Mechanistically, the excessive reactive oxygen species were commonly derived from the accumulation of advanced glycosylation, increased polyol pathway flux and the mitochondrial dysfunction caused by the excessive activity of hexosamine pathway^33^. According to our results, the production of ROS showed a sharp rise in diabetic renal tissues, whereas ISL clearly neutralized the over-generation of ROS (Fig. 3e).

Nrf-2 has been considered as an effective regulator of antioxidative genes expression, for example, HO-1 and NQO-1, and in modulating ROS-eliminating enzymes^34^. Many natural compounds with antioxidative properties, such as honokiol^35^, notoginsenoside^36^, and piceatannol^37^, could facilitate the treatment of diabetic complications by activating Nrf-2-mediated signaling pathway. Recently, Yerra et al.^16^ demonstrated that promoting Nrf-2-mediated antioxidative response could mediate the protective effects of ISL on diabetic neuropathy. Herein, it was discovered that ISL exerted renal protection by activating Nrf-2, followed by the upregulation of HO-1 expressions (Figs. 3f–h, 4d, i, j), and finally the downregulation of ROS generation under diabetic condition. In summary, The beneficial actions of ISL are closely associated with its property to inhibiting HG-induced oxidative stress via increasing Nrf-2.

SIRT1 is significant to the pathogenesis and development of DN^38^. The upregulation of SIRT1 suppresses DN in various diabetic animal models and in renal cells, including renal proximal tubular cells, podocytes, and mesangial cells^3^. By reducing inflammation, oxidative stress, and modulating metabolic homeostasis, SIRT1 produces renoprotective effects^39^. Mechanistically, acetyl groups were removed by SIRT1 as a NAD+-dependent deacetylase from the proteins involved in DN^3^. Having been defined as an activator of SIRT1, several natural compounds and synthetic agents can upregulate its expression and activity, thus protecting against DN. Notably, as indicated by Yerra et al.^16^, ISL could activate SIRT1 to reduce diabetic neuropathy. According to the molecular docking results obtained in this study, ISL could bind to SIRT1 with Thr-209, Glu-214, Asp-292, Asp-298, Val-412, Asn-417, Leu-418, His-423, Lys-444, and Arg-446 amino acids. A variety of studies in diabetic complications have also demonstrated that SIRT1 can serve as an upstream regulator for Nrf-2^35,40,41^, as well as p38 MAPK signaling pathway^42–44^. Meanwhile, it has been determined that the antiinflammatory and antioxidant properties of ISL are dependent on SIRT1, as shown in Fig. 6. Notably, ISL reversed the activation of p38 and depletion of Nrf-2 by activating SIRT1, suggesting a possibility that the SIRT1-p38-Nrf-2 pathway is the underlying mechanism behind the inflammation and oxidative stress in DN.

In summary, our findings revealed that ISL was highly effective in protecting against the progression of DN by reducing inflammatory response and oxidative stress. Mechanistically, SIRT1 mediated the effectiveness of ISL to lead to the improvement of downstream p38 MAPK-mediated inflammatory and Nrf-2-mediated oxidative signaling pathways. Considering these favorable activities, a conclusion could be drawn that the new natural flavonoid compound ISL has a massive potential to be applied to alleviate hyperglycemia-induced renal injuries in clinic practice. However, a limitation of this study is that we only used mouse model of STZ-induced type 1 DN. As we know, other types of nephropathy were clinically common and refractory, such as type 2 DN, LPS-induced acute kidney injury, hypertensive nephropathy, and obstructive nephropathy. Protective effects of ISL will be fully explored for renal injuries in future studies.

## Materials and methods

### Reagents and chemicals

Glucose was obtained from Sigma (St. Louis, MO). ISL (PubChem CID: 638278) was sourced from Aladdin (Shanghai, China). The compound ISL dissolved in dimethyl sulfoxide and 0.5% sodium carboxyl methyl cellulose (CMC-Na) during in vitro and in vivo experiments, respectively. Antibodies against (TGF-β, sc-130348), Bcl-2-like protein 4 (Bax, sc-7480), B-cell lymphoma 2 (Bcl-2, sc-7382), extracellular signal-regulated kinase (ERK, sc-514302), phosphorylated ERK (p-ERK, sc-7383), Nrf-2 (sc-365949) and heme oxygenase-1 (HO-1, sc-136960) were procured from Santa Cruz Biotechnology (Santa Cruz, CA, USA). Antibodies against c-Jun N-terminal kinases (JNK, 9252 S), phosphorylated JNK (p-JNK, 4668 S), P38 (9212 S), phosphorylated P38 (p-P38, 9211 S), Cl-C3 (cleaved caspase 3, 9664), and glyceraldehyde-3-phosphate dehydrogenase (GAPDH, 5174 S) were obtained from Cell Signaling (Danvers, MA, USA). Antibodies against collagen type 4 (Col-IV, ab6586), TNF-α (ab1793), macrophage marker F4/80 (ab6640), and 3-nitrotyrosine (3-NT, ab61392) were obtained from Abcam (Cambridge, MA, USA). Horseradish peroxidase-conjugated anti-rabbit secondary antibodies were obtained from Santa Cruz Biotechnology (sc-2357). One-step TUNEL apoptosis assay kit was obtained from Beyotime (Beijing, China).

### In vitro studies

Rat renal tubular epithelial cell line (NRK-52E) were purchased from Shanghai Institute of Biochemistry and Cell Biology (Shanghai, China) for growth at 37 °C. They were maintained at 5% CO_2_ in Dulbecco’s Modified Eagle’s Medium (DMEM) (Gibco, Eggenstein, Germay) containing 5.5 mM of d-glucose supplemented with 10% fetal BSA, 100 U/mL of penicillin, and 100 mg/mL of streptomycin. In the experimental HG group, the cells were cultured with DMEM medium containing 33 mM glucose.

### In vivo studies

The sample animals were 28 male C57BL/6 mice aged 6–8 weeks, as obtained from the Animal Center of Wenzhou Medical University (Wenzhou, China). Housed at a constant room temperature, the mice were subject to a 12:12 h light–dark cycle, and fed with a standard rodent diet and water. In line with the ‘The Detailed Rules and Regulations of Medical Animal Experiments Administration and Implementation’ (Document No. 1998-55, Ministry of Public Health, PR China), the protocols of animal experiments were granted approval from the Animal Policy and Welfare Committee at Wenzhou Medical University (Approval Document no. wydw2016-0130). Blinded experimenters were deployed to conduct all animal experiments. The assignment of treatment groups was carried out on a random basis.

To induce the T1DM model, 8-week-old mice were subjected to intraperitoneal injection with 50 mg/kg STZ (Sigma-Aldrich) for a 5-day period, while control animals (*n* = 7) were given citrate buffer of the identical volume. On days 3 and 7, blood glucose levels of the mice in both conditions were monitored with a glucometer. One week after the STZ injection, the mice with fasting-blood glucose level exceeding 12 mM were regarded diabetic for further study. The resulting mice were then assigned into three groups on a random basis: diabetic mice (STZ-DM1, *n* = 7), ISL low concentration-treated DM (STZ-DM1 + ISL-10, *n* = 7) and ISL high concentration-treated DM (STZ-DM1 + ISL-20, *n* = 7). In the DM + ISL-10 group and DM + ISL-20 group, mice were treated with the oral administration of 10 or 20 mg/kg ISL every 2 days. In contrast, with the same schedule followed, mice (*n* = 7) received 0.5% CMC-Na solution only in the DM group and age-matched control group. Following ISL administration, the level of body weight and blood glucose levels was logged on a weekly basis. The treatment lasted for 12 weeks, all animals were killed after anesthesia. In all, 24 h before sacrifice, mice urine was obtained. At this time, the body and renal weight were logged. The serum and renal tissues were collected and stored at −80°C for further analysis.

Measurement of urine protein, SCr, BUN in serum. In line with the instructions from the manufacturer, commercial kits (Nanjing Jiancheng, Jiangsu, China) were applied to measure the levels of urine protein, SCr, and BUN.

### H&E and PAS staining

Renal tissues were fixed in 4% paraformaldehyde solution, embedded in paraffin and cut into 5 μm sections. Then, the sections were dehydrated and subject to hematoxylin and eosin staining. In order to assess histopathological damage, an image was of the sections was captured, with each of them observed with the assistance of a light microscope (×400 amplification; Nikon, Japan).

### Masson and Sirius Red staining

Subsequent to deparaffinization and rehydration, the paraffin sections (5 μm) of kidney specimens were stained for connective tissue with Masson’s trichrome staining in line with the instructions from the manufacturer. In addition, 0.1% Sirius Red and 1.3% saturated aqueous solution of picric acid were applied to stain the sections for evaluating the deposition of type IV collagen. All images were taken with an epifluorescence microscope fitted with a digital camera using its bright-field illumination function (Nikon, Japan).

### Immunochemistry for TNF-α and 3-NT detection

Xylene was applied to isolate the paraffin samples (5 μm) from the sections, after which graded alcohol series were used to hydrate the samples again. Subsequently, with microwave applied, antigen was retrieved on the deparaffinized sections in 0.01 mol/l citrate buffer (pH 6.0). Afterwards, they were placed in 3% hydrogen peroxide in methanol at room temperature for 30 min, which was followed by blocking with 5% BSA and incubation with anti-3-nitrotyrosine (NT) antibody (1:500) and anti-TNF-α antibody (1:500) at 4 °C overnight and the subsequent addition of the secondary antibody (1:200; Santa Cruz Biotechnology). A 3,30-diaminobenzidine solution was applied to visualize the reaction. Then, the sections were counterstained with hematoxylin, dehydrated and observed under a fluorescence microscope (×400 amplification; Nikon).

### Immunofluorescence for F4/80 and ROS detection

For the purpose of immunofluorescence observation, the 5 μm kidney sections were subject to deparaffinization and rehydration. After being treated with 3% H_2_O_2_ for 30 min, the kidney sections were treated with 1% albumin from bovine serum (BSA) in phosphate-buffered solution (PBS) for another 30 min. Slides were incubated with anti-F4/80 antibody (1:100) at 4 °C overnight, which was followed by the incubation with HRP secondary antibody at room temperature for 1 h. After being incubated in PBS containing 2 μM DHE at 37 °C for 2 h, the slides were washed three times. 4′,6-diamidino-2-phenylindole was applied for detection and hematoxylin for the visualization of nuclei. A fluorescence microscope was used to observe the images.

### TUNEL staining

In line with the manufacturer’s protocol, 5 μm sections derived from the kidney tissues were used for TUNEL apoptosis detection kit (R&D Systems, Minneapolis, MN). A fluorescence microscope (×400 amplification; Nikon Tokyo, Japan) was used to observe TUNEL-positive cells.

### Real-time quantitative PCR (RT-qPCR)

The Trizol reagent (Invitrogen, Carlsbad, CA, USA) was applied to extract total RNA from NRK-52E or homogenized kidney tissue samples (10–20 mg). The mRNA level analysis of MCP-1, IL-6, Nrf-2, HO-1, COL-IV, CTGF, TGF-β, and β-actin was conducted with a prior report as reference. The primer sequences are shown in Supporting Information Table S1.

### Western blot

Radioimmunoprecipitation assay lysis buffer (BOSTER biological technology, Wuhan, China) was used to extract total protein from cells or tissue samples. Every group of tissue sample contained at least three kidney tissue extracts, each from a different mouse. The lysates of three kidney tissues were separated with sodium dodecyl sulfate polyacrylamide gel electrophoresis, before the electronic transferral to a nitrocellulose membrane. Each of the membranes was subject to pre-incubation in tris-buffered saline (pH = 7) containing 0.05% Tween 20 and 5% non-fat milk at room temperature for 1.5 h. Afterwards, they were subject to incubation with specific antibodies at 4 °C overnight. The immunoreactive bands were discovered after incubation with secondary antibody conjugated with horseradish peroxidase, while the bands were visualized using enhanced chemiluminescence reagents. Image J analysis software was utilized to analyze the amount of proteins, which was then normalized to GAPDH.

### Molecular docking

The potential binding mode between ISL-SIRT1 was calculated using the multiple ligand simultaneous docking software^45^. The human SIRT1 structure was retrieved from the Protein Data Bank (PDB) database (PDB code: 5BTR)^21^. The UCSF Chimera software was employed to preprocess the SIRT1 structure in a series of steps by removing all water molecules and hetero-atoms and adding hydrogen atoms^46^. The binding site was covered with a grid box sized 22.5 Å × 22.5 Å × 22.5 Å. Afterwards, the ISL at different ratios was docked into the binding site of SIRT1. During molecular docking, 100 conformations were obtained and then scored using the PSO method. The docking energies were subsequently ranked, with the optimal conformations considered for further analysis.

### SIRT1 siRNA transfection and gene silencing

SIRT1 siRNA was derived from Gene Pharma (Shanghai, China), as well as the scramble siRNA which was used as a negative control. In line with the manufacturer’s protocol, Lipofectamine 2000 (Invitrogen, CA, USA) was used for NRK-52E cells to be transiently transfected with 100 nM siRNA target sequences or negative control siRNA. After 4–6 h of incubation, the cells were washed and fresh medium was added. The transfected cells were subject to HG treatment for the subsequent studies. The transfection efficiency was finally confirmed by western blotting.

### SIRT1 plasmid transfection-induced and gene overexpressing

To overexpress SIRT1 in NRK-52E cells, the cells were transfected with pAd-Track-SIRT1 plasmid or pAd-Track vector as a control (Addgene, Cambridge, MA), with Lipofectamine 3000 as manufacturer instructed (Invitrogen, USA). After 4–6 h of incubation, the cells were washed and fresh medium was added. The transfected cells were subjected to HG treatment for later research. Western blotting was performed to confirm the transfection efficiency.

### Statistical analysis

The data were obtained from four independent experiments for in vitro studies and seven mice in every experimental group for in vivo studies. In each experiment, the values obtained were subject to a normality test and the values for statistical analysis (*n* = 4) conformed to normal distribution. All data were indicated by means ± SDs. The analysis of variance was conducted in GraphPad Pro 6.0 (GraphPad, San Diego, CA) to compare different groups, with a statistically significant result obtained at *p* < 0.05.

## Supplementary information

Supplementary Figure 1

Supplementary Figure 2

Supplementary Figure 3
