# Supplementary figures and images for: Isoliquiritigenin prevents hyperglycemia-induced renal injuries by inhibiting inflammation and oxidative stress via SIRT1-dependent mechanism
Source: Cell Death Dis. 2020 Dec 7;11(12):1040. doi: 10.1038/s41419-020-03260-9 (PMC7721869; doi:10.1038/s41419-020-03260-9)

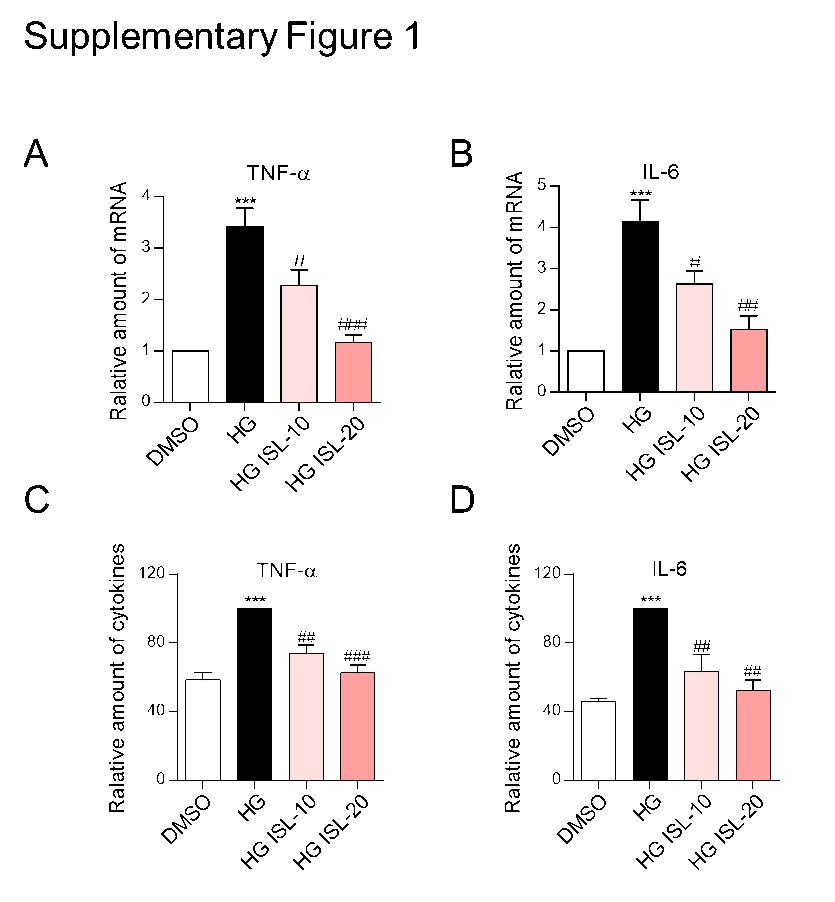

Supplement: Supplementary file 1 — Supplementary Figure 1 [file 41419_2020_3260_MOESM1_ESM.png]

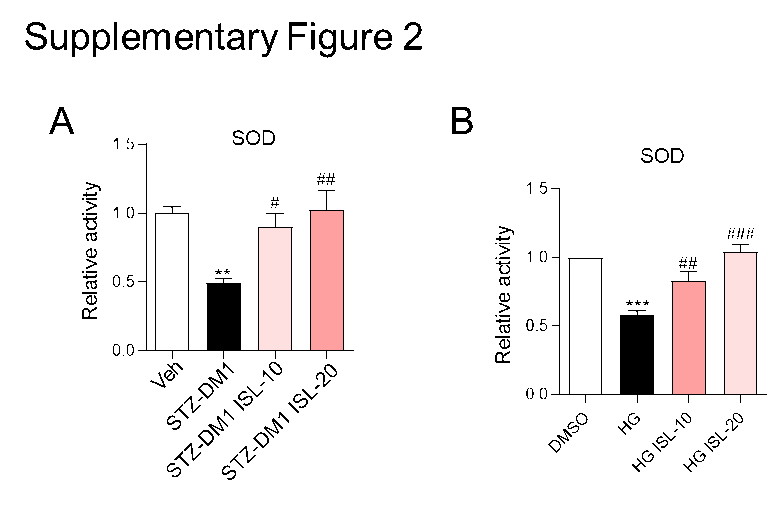

Supplement: Supplementary file 2 — Supplementary Figure 2 [file 41419_2020_3260_MOESM2_ESM.png]

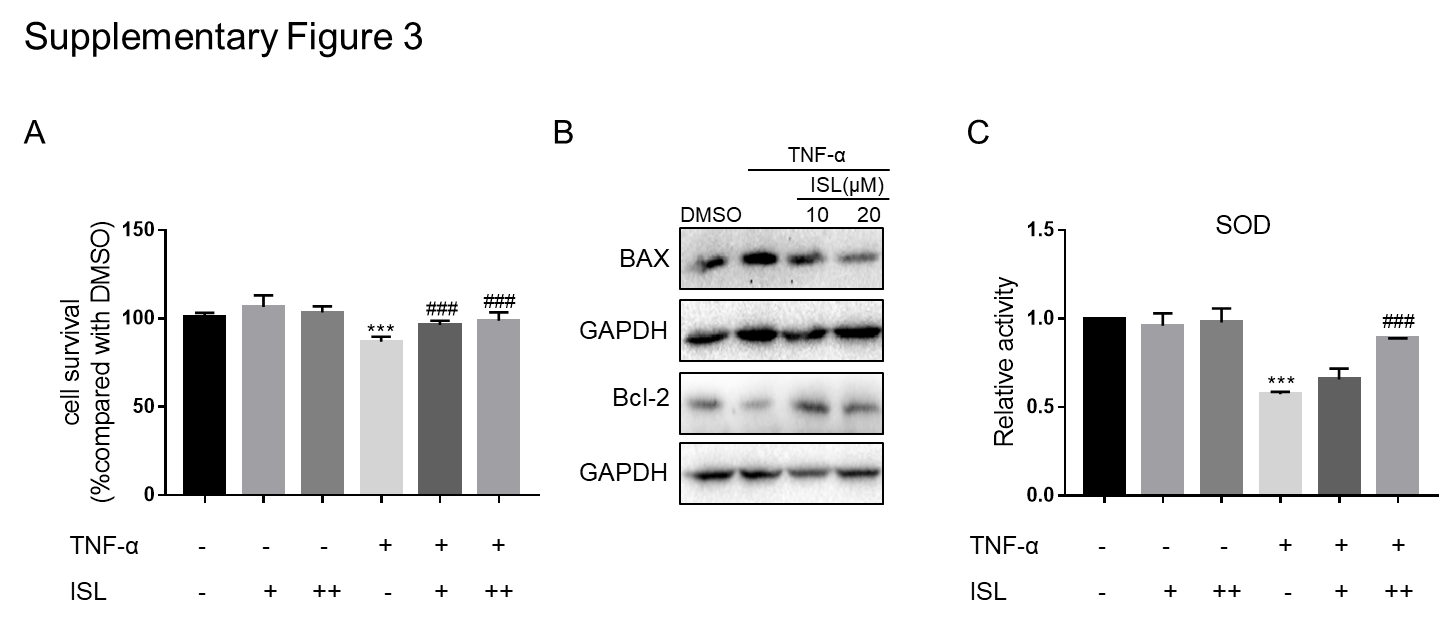

Supplement: Supplementary file 3 — Supplementary Figure 3 [file 41419_2020_3260_MOESM3_ESM.png]
